# Population Recovery of Nicobar Long-Tailed Macaque *Macaca fascicularis umbrosus* following a Tsunami in the Nicobar Islands, India

**DOI:** 10.1371/journal.pone.0148205

**Published:** 2016-02-17

**Authors:** Avadhoot D. Velankar, Honnavalli N. Kumara, Arijit Pal, Partha Sarathi Mishra, Mewa Singh

**Affiliations:** 1 Sálim Ali Centre for Ornithology and Natural History, Coimbatore, Tamil Nadu, India; 2 Manipal University, Manipal, Karnataka, India; 3 Biopsychology Laboratory and Institution of Excellence, University of Mysore, Mysore, Karnataka, India; 4 Organismal Biology Unit, Jawaharlal Nehru Centre for Advance Scientific Research, Bangalore, Karnataka, India; 5 National Institute of Advance Studies, Bangalore, Karnataka, India; National Cheng-Kung University, TAIWAN

## Abstract

Natural disasters pose a threat to isolated populations of species with restricted distributions, especially those inhabiting islands. The Nicobar long tailed macaque.*Macaca fascicularis umbrosus*, is one such species found in the three southernmost islands (viz. Great Nicobar, Little Nicobar and Katchal) of the Andaman and Nicobar archipelago, India. These islands were hit by a massive tsunami (Indian Ocean tsunami, 26 December 2004) after a 9.2 magnitude earthquake. Earlier studies [Umapathy et al. 2003; Sivakumar, 2004] reported a sharp decline in the population of *M*. *f*. *umbrosus* after thetsunami. We studied the distribution and population status of *M*. *f*. *umbrosus* on thethree Nicobar Islands and compared our results with those of the previous studies. We carried out trail surveys on existing paths and trails on three islands to get encounter rate as measure of abundance. We also checked the degree of inundation due to tsunami by using Normalized Difference Water Index (NDWI) on landsat imageries of the study area before and after tsunami. Theencounter rate of groups per kilometre of *M*. *f*. *umbrosus* in Great Nicobar, Little Nicobar and Katchal was 0.30, 0.35 and 0.48 respectively with the mean group size of 39 in Great Nicobar and 43 in Katchal following the tsunami. This was higher than that reported in the two earlier studies conducted before and after the tsunami. Post tsunami, there was a significant change in the proportion of adult males, adult females and immatures, but mean group size did not differ as compared to pre tsunami. The results show that population has recovered from a drastic decline caused by tsunami, but it cannot be ascertained whether it has reached stability because of the altered group structure. This study demonstrates the effect of natural disasters on island occurring species.

## Introduction

Natural disasters may pose a serious threat to animal populations [1]. Survival of a population after a disastrous event is dependent on plasticity of the species to sudden changes in its population and habitat [2]. The plasticity, dependent on evolutionary history and environment of a species [3], decides the minimum population size necessary for the species to recover. The habitat specialists or the low occurrence species are more risk prone to extinction than the species occurring in large populations or with wide distributions [3]. One of the important environmental determinants of plasticity is the severity of impact and duration of a disaster[4]. Thus, natural disasters can be classified into three categories: 1) High severity with short duration (e.g. Hurricanes, tsunami, fire, and disease outbreaks), 2) low severity with long duration (e.g. Drought, climate change), and 3) high severity with cyclic occurrence (e.g. Floods, El Niño event).However, some natural disasters, such as disease outbreaks, which may eliminate a population, have no impact on the structure of its habitat. On the other hand, disasters like the tsunami may affect the habitat and the animal populations both.

There are two kinds of mortalities based on the effects induced by natural disasters; primary and secondary. Primary mortality includes animals dying directly due to natural disasters while secondary mortality occurs due to changes in habitat structure leading to thedeath of vulnerable individuals [5]. For example, increased mortality of juveniles and cohorts of various ottariids (seals) due to over harvesting and the El Niño event [4], increased mortality of infants, juveniles and females in *Lemur catta* due to drought [6], and breeding failure in *Aptenodytes patagonicus* due to tsunami [7]. Such differential mortality indeed alters the group composition in a population, affecting its recovery. Recovery is also governed by behaviour and complexity of social systems of a species. For instance, a species with cryptic mating rituals or acomplex social system becomes more vulnerable to extinction due to these effects. It is more so with group living species thathave cooperative feeding and defence. Thus, such secondary mechanism reduces overall inclusive fitness of the population and reduces its rate of recovery leading to ‘Allee effect’ and possibilities of multiple Allee effects [8,9]. Although the prevalence and strength of these effects are poorly known, studies report these effects on a variety of species, for example *Marmota marmota* occurring in low densities have difficulties in finding mates and their survival is decreased due to less social thermo-regulation [9], and *Laytonictus* faces reduced breeding capacity, reduced anti-predatory response and reduced cooperative hunting [9].Such mechanisms increase the stochasticity of population dynamics making it harder to predict population trends during recovery. Predicting population trends of animal groups such as primates is particularly difficult because of such high demographic stochasticity which may be due to their social and behavioural complexity. Primates react differently to various disasters. For instance, *Allouata paliata* recovered, 3 decades after a suspectedyellow fever outbreak [10], but it showed varied response to regenerating forest [11]. *M*. *fascicularis* showed behavioural flexibility in response to severely altered environment [12]. Apart from such instances, another limiting factor to population recovery is the rate of recolonizaton of unoccupied habitats by forming new groups. This happens by fission of large neighbouring groups or by dispersed individuals jointly occupying unoccupied patches [11,13].

Studies report the immediate impacts of tsunamis on various terrestrial and marine fauna, for example breeding failure in *Aptenodytes patagonicus* at Crozet Archipelago [7], reduction in the abundance of sea birds at Latham Island, Tanzania [14]and short term changes in distribution and abundance of *Sephanoides fernandensis* at Robinson Crusoe Island, Chilean coast[15]. Similarly, some immediate impacts of the tsunami on fauna in Andaman and Nicobar islands were reported; for example sharp declines in populations of *Megapodius nicobarensis* and *M*. *f*. *umbrosus* [16,17], increased population size of fish *Chanos chanos*[18]and loss of nesting grounds for marine turtles due to submergence of sandy beaches [19].Many such studies put emphasis on vulnerability of islands to natural disasters. Such vulnerability of an island depends on size of the island. It has been observedthat smaller islands suffer overwhelming natural and socioeconomic impacts compared to continents dueto natural disasters [20]. Small islands have limited habitats which in turn governs distribution of species making them susceptible to extinction. This was observed in GwaiiHaanas archipelago, where distribution of various bird species was dependent on old growth forests which were correlated with island size and its isolation [21].

Macaques are highly adaptable primates to changing habitat [12], and live in all possible habitat types and environmental conditions [22,23]. *M*. *fascicularis* is widely distributed throughout south-east Asian countries. *M*. *fascicularis* inhabits urban environments as well as interior forests[24], however, it is commonly found along seashore, mangrove forests and swamps[25–28]making it more vulnerable to atsunami. *M*. *fascicularis* is also considered as a wide spread but rapidly declining species[29].

The subspecies of long-tailed macaque in India is *M*. *f*. *umbrosus*, which is endemic to the three Nicobar Islands including Great Nicobar, Little Nicobarand Katchal[30].It has been categorized as ‘Vulnerable’ in IUCN Redlist[31]due to its restricted distribution, and susceptibility of its habitat to natural calamities. The subspecies is accorded the highest protection under ‘Schedule-I’ of the Indian Wildlife Protection Act[32].The 2004 Indian Ocean tsunami destroyed much of the coastal habitat, and even the inland forests in some of the areas [33,34]. *M*. *f*. *umbrosus*[16,30]is the only species which was assessed using thesame methodologies before and after thetsunami [16,30], and asharp decline in the *M*. *f*. *umbrosus* numbers in all threeislands was reported[16].Thus, we selected *M*. *f*. *umbrosus* as a suitable species to study the long term response to a sharp decline in their population size due to a tsunami in an island environment. We presumed that the impact of the tsunami would be localized to coastal regions, and that, over the time inland population of *M*. *f*. *umbrosus* thatwasunaffected would recolonize coastal regions. Further, the extent of damage to the habitat and population was expected to be more on the smaller islands than on the larger islands. We examined the current status of *M*. *f*. *umbrosus* in the Nicobar group of islands using thesame field protocol as that of Umapathy et.al. [30]and Sivakumar[16]to understand the long-term response of the species that had declined sharply.

## Methods

### Study Area

**Fig 1:**
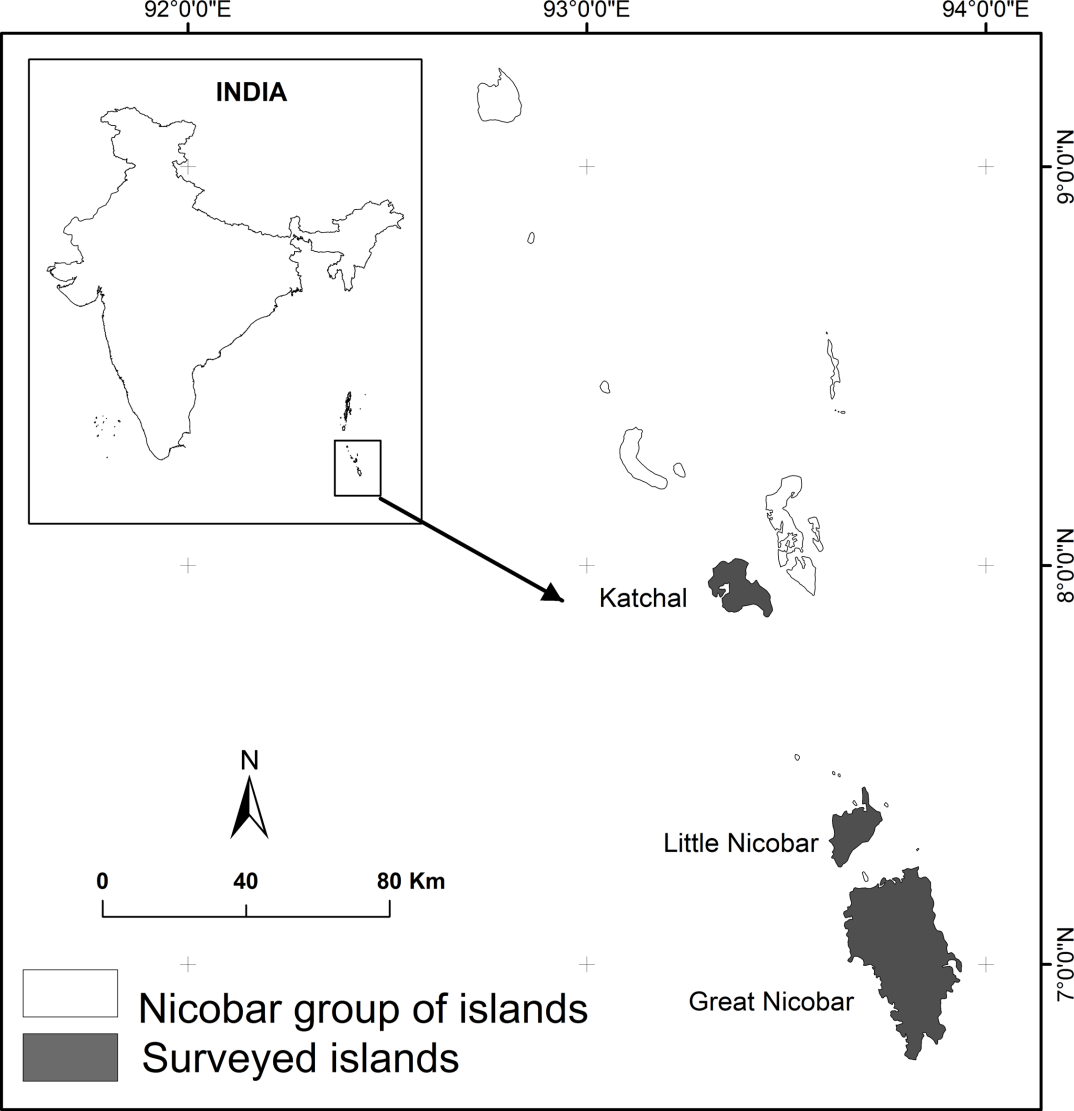
Map of study sites in Nicobar group of islands.

The present study was conducted at Great Nicobar, Little Nicobar and Katchal lying between 93° 38ʹ 05.6″- 93° 57ʹ 13.7″ E and 6° 44ʹ 7.8″- 7° 13ʹ 46.6″ N, 93° 36ʹ 14.0″-93° 46ʹ 17.4″ E and 7° 14ʹ 45.2″- 7° 26ʹ 33.7″ N, 93° 28ʹ 32.9″-93° 18ʹ 06.8″ E and 7° 52ʹ 24.2″-8° 1ʹ 33.6″ N respectively (Fig 1). The current island size of Great Nicobar, Little Nicobar and Katchal is 895.48, 138.25 and 139.39 km^2^ respectively. The highest elevation in the Great Nicobar, Little Nicobar and Katchal islands is 642 m, 470 m, and 230 m ASL respectively. The major vegetation types in the islands include littoral beach formations and mangrove vegetation on coastal regions, and inland vegetation is characterized by evergreen hill forests and low land swamps of *Pandanus* and *Areca* species [35]. Apart from natural vegetation these islands also have manmade coconut plantations, and plantations of other species introduced for food or ornamental value[35]. Themassive upheaval was caused by an earthquake before the tsunami and had severely damaged the coastal vegetation of the Nicobar group of islands[34].

### Survey design and Methodology

We surveyed *M*. *f*. *umbrosus* in the three islands between January 2013 and April 2014. Since the undergrowth in the forest was very thick, we used a trail survey like Umapathy et. al. [30]. Further, heavy tree fall due to thetsunami made most part*s* of the islands inaccessible for distance sampling. Therefore, the existing trails and road network werechosen to survey *M*. *f*. *umbrosus*. Pre fixed trails were chosen, and the area was walked between 0700 and 1100 hoursat an average speed of 1km per hour. At every sighting of amacaque group, geographical position was recordedusing a handheld GPS (GarminGPSmap 76CSx).In the case of direct sightings inclose proximity, group size, age and sex of the visible individuals were also recorded. Group counts were taken when group*s* were crossing clearings such as roads, trails, streams or narrow open areas. In case all individuals of a group were found to be moving in same direction, macaques intercepting an imaginary line from a vantage point were counted using tally marks for each age and sex class.

Forest Types: Basic habitat types and dominant vegetation were noted at each sighting of a group during thepopulation status and distribution survey. During thetrail survey, vegetation parameters were recorded with change in habitat types. Major forest types were distinguishedbased on vegetation composition[35].

### Analysis

To detect post tsunami changes in shoreline, we compared current Landsat 8 imageries with 30m spatial resolution (Dated 12- April– 2014 for Great Nicobar, 2-March-2014 for Katchal and Little Nicobar) (downloaded from www.earthexplorer.com, U.S. Geological Survey) with pre-tsunami Landsat 7 imageries (Dated 5- March -2003 for Great Nicobar and Little Nicobar, 23 –Jan– 2003 for Katchal). To extract shorelines from the images, we used Normalized Difference Water Index (NDWI) [36], which segregated land and water regions from the images. The current shoreline for the three islands was extracted by converting NDWI raster images to vector formats. For pre-tsunami shoreline, cloud free data was not available, but the visible portion of shoreline of pre-tsunami NDWI raster matched closely with the vector map downloaded from DIVA GIS (divagis.org), hence those vector maps were used as reference pre-tsunami shoreline. Pre tsunami and post tsunami coastlines obtained thus were used to find out net change in shoreline, degree of inundation and total area submerged. Equidistant parallel transects intersecting both pre and post tsunami coastlines were overlaid from an offshore baseline. Distance between consecutive transects was kept at 250 m., intersecting length of each transect in between two shorelines was obtained to estimate degree of inundation. Comparisons for degree of inundation were made between the islands and between west coast and east coast regions of each island.

For processing images and basic GIS operations, we used Quantum GIS (QGIS v2.4.0), while net change in shore line and degree of inundation was computed using Digital Shoreline Analysis System (DSAS) extension in ArcGIS 10[37].

The abundance of *M*. *f*. *umbrosus* is represented as encounter rate (groups per kilometre) obtained by dividing the number of groups by the length of transects in each place. To compare differences in encounter rate of *M*. *f*. *umbrosus* between islands, habitat types and before and after tsunami, we employed generalized linear model (GLM) with Poisson distribution.GLM was run with number of groups as response variable with log transformed trail length as offset parameter. Islands, habitat types and period were set as categorical explanatory variables. Chi square test was used to compare the proportions of males between islands, and abundance of *M*. *f*. *umbrosus* before and after tsunami respectively.Independent sample student t-test and one way ANOVA were used to compare the mean group size and age-sex ratios between the islands and for before and after tsunami respectively. Overall rate of change in population was computed using intrinsic rate of change [38]. All statistical analysis was carried out using R statistical language V 3.02 with R Studio IDE for R v0.98.953.

### Ethical Note

All guidelines and regulations of the country of the study area were adhered to while conducting this research. TheStudy was also approved by theethical committee of Sálim Ali Centre for Ornithology and Natural History (SACON), Coimbatore, prior to its commencement. All necessary permits to enter protected area and tribal area were acquired from Chief Wildlife Warden of Andaman and Nicobar Forest Department and, Assistant Commissioner Andaman and Nicobar Administration (Permit No. CWLW/WL/134/566).

## Results

### Current population status

#### Abundance

**Fig 2:**
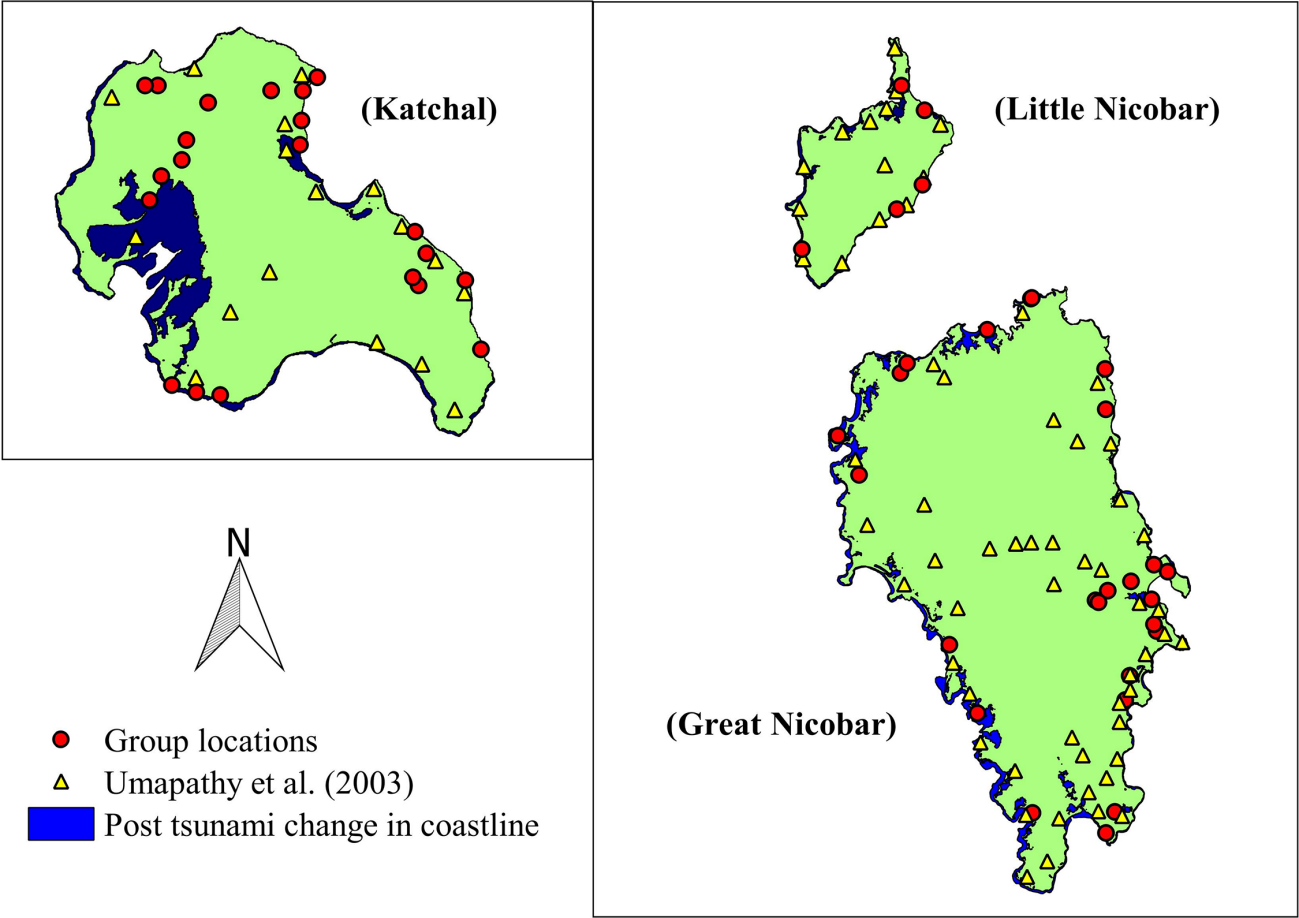
Map of Nicobar Islands depicting inundated area due to tsunami and group location of current study with group locations of pre-tsunami study.

We sighted 36, 5 and 38 *M*. *f*. *umbrosus* groups during the 119.55, 14.09 and 78.50 km of trail surveys in Great Nicobar, Little Nicobar and Katchal respectively (Table 1, Fig 2). The group encounter rate per kilometre in Great Nicobar (0.30) was significantly lower than in Katchal (0.48) (Walds Z = 2.03, p = 0.04) with no difference betweenLittle Nicobar (0.35)andthe other two islands (Table 1). The group encounter rate in Plantation (0.58) was significantly higher than in Evergreen (0.28) (Walds Z = 2.92, p = 0.004) and Littoral forest (0.17) (Walds Z = 2.38, p = 0.02) with no difference between Mixed forest (0.49) and other forest types (Table 2). Similarly,group encounter rate in Coastal habitat (0.49) was significantly higher than in Inland habitat (0.31) (Walds Z = 2.05, p = 0.04) (Table 2).

**Table 1:** Mean number of *M*. *f*. *umbrosus* groups encountered per kilometre in Nicobar Islands.

Island	No of trails	Mean trail length ±SD, (min-max)	Total effort (km)	Groups detected n	n groups/km
Great Nicobar	26	4.59 ± 7.28 (0.3–34.12)	119.55	36	0.30
Little Nicobar	9	1.57 ± 0.96 (0.37–4.20)	14.09	5	0.35
Katchal	21	4.90 ± 2.70 (0.34–16.75)	78.50	38	0.48
Overall	56	2.52 ± 4.32 (0.34–34.12)	212.14	79	0.37

**Table 2:** Mean number of *M*. *f*. *umbrosus* groups encountered per kilometre in different forest and habitat type in Nicobar Islands.

Type	Overall (of all the three islands)			
	n trails	Groups	Km	n groups/km
**Forest Type**				
Evergreen hill forest	22	32	111.48	0.28
Mixed forest	10	9	18.25	0.49
Plantation	13	34	58.32	0.58
Littoral forest	11	4	23.88	0.17
**Habitat Type**				
Inland	28	44	141.54	0.31
Coastal	28	35	70.59	0.49

#### Population characteristics

During the study period reliable group counts and demography information on little Nicobar island could not be recorded, hence data of only Katchal and Great Nicobar islands was compared. The mean group size between Great Nicobar (39.83±17.47, N = 6) and Katchal (43.50±26.15, N = 4)(Table 3) did not vary significantly (M-W U test, U = 12.50, p = 0.91). Percent adult males, adult females and immature per group between Great Nicobar and Katchal (adult males: χ^2^ = 5.88, df = 10, p = 0.82; adult females: χ^2^ = 5.31, df = 10, p = 0.86; immature: χ^2^ = 5.18, df = 10, p = 0.87) also did not vary significantly (Table 4). Although, the number of females per male in the Katchal (1.92±0.63) was less than in Great Nicobar (2.59 ±0.91), it did not differ significantly (t = -1.285, df = 8, p = 0.235). However, the number of immature*s* per adult was significantly higher (t = -3.171, df = 8, p< 0.01) in Katchal (1.94 ±0.32) than in Great Nicobar (1.01±0.52).

**Table 3:** Group Size and age-sex composition of *M*. *f*. *umbrosus* groups in Nicobar Islands.

Island	Group	Adult Male	Adult Female	Sub-adult + Juvenile	Infant	Total
Great Nicobar	B quarry group	6	13	20	6	45
Great Nicobar	Temple group	5	7	8	0	20
Great Nicobar	Laxman beach group	4	10	16	8	38
Great Nicobar	Chinganbasti group	5	13	7	4	29
Great Nicobar	09 Km N-S Road	7	19	8	2	36
Great Nicobar	20 Km N-S Road	6	25	28	12	71
Katchal	Sea wall group	5	7	17	7	36
Katchal	Kapanga group	3	4	8	3	18
Katchal	Oil palm I	7	17	36	20	80
Katchal	Oil palm II	4	10	22	4	40

**Table 4:** Group structure and age-sex ratio of *M*. *f*. *umbrosus* in Nicobar Island

Island	% Males (±SD)	% Females (±SD)	% Immature (±SD)	Ad ♂: Ad♀	Ad: Imm	Ad♀: Inf
Great Nicobar	15.67 (±6.13)	37.17 (±9.97)	47.16 (±13.89)	2.59 (±0.91)	1.01 (±0.52)	3.65 (±3.35)
Katchal	12.33 (±3.63)	21.98 (±2.32)	65.69 (±3.70)	1.92 (±0.63)	1.94 (±0.32)	1.42 (±0.75)

Ad ♂ = adult males, Ad♀ = adult Females, Imm = immatures, Inf = infants.

### Impact of 2004 tsunami

#### Spatial extent of damage

Rate of shoreline ingress was more on thewestern coasts of all three islands and the eastern coasts received relatively littledamage. The estimated total inundated area using shoreline change due to the 2004 tsunami in Great Nicobar, Little Nicobar and Katchal was 51.91, 10.04 and 21.80 km^2^ respectively, thus the current island size is 895.48, 138.25 and 139.39 km^2^ respectively (Table 5).

**Table 5:** Coast line variation after 2004 December tsunami in Nicobar Islands.

Island	Mean Ingress (m)	Max (m)	Inundated area (km^2^)	Pre tsunami area	% area submerged
Great Nicobar	228.34	1926.0	51.91	947.39	5.48
Little Nicobar	293.97	1751.0	10.04	148.29	6.77
Katchal	407.21	2800.0	21.80	161.19	13.52

#### Change in the population

The group encounter rate during the three surveys carried out in 2000, 2006 and 2014 (Table 6) in the three islands was likely to be significantly lower in 2006 (0.10) than in 2000 (0.23) (Walds Z = 4.19, p = 0.001) and 2014 (0.37) (Walds Z = 6.60, p = 0.001). Also, the group encounter rate in 2014 was significantly higher than in 2000 (Walds Z = 3.11, p = 0.002). The population increased at an intrinsic rate (*r*) of 0.12, 0.14 and 0.17 in Great Nicobar, Little Nicobar and Katchal from 2006 to 2014 (Table 6).

**Table 6:** Number of *M*. *f*. *umbrosus* groups encountered per kilometre during 2000, 2006 and 2014 studies in Nicobar Islands.

Island	Pre Tsunami			Post Tsunami						Intrinsic rate *r* (2006–2014)
	2000			2006			2014			
	n trails (Km)	nGroups	groups/km	n trails (Km)	n Groups	n groups/km	n trails (Km)	n Groups	n groups/km	
Great Nicobar	16 (227.9)	53	0.23	41 (211.8)	22	0.104	26 (119.5)	36	0.30	0.12
Little Nicobar	12 (62.7)	17	0.27	23 (99.1)	10	0.101	9 (14.1)	5	0.35	0.14
Katchal	12 (92.5)	18	0.19	17 (76.5)	8	0.105	20 (78.5)	38	0.48	0.17
Overall	40 (383.1)	88	0.23	81 (387.4)	40	0.103	51 (212.1)	79	0.37	0.14

**Fig 3:**
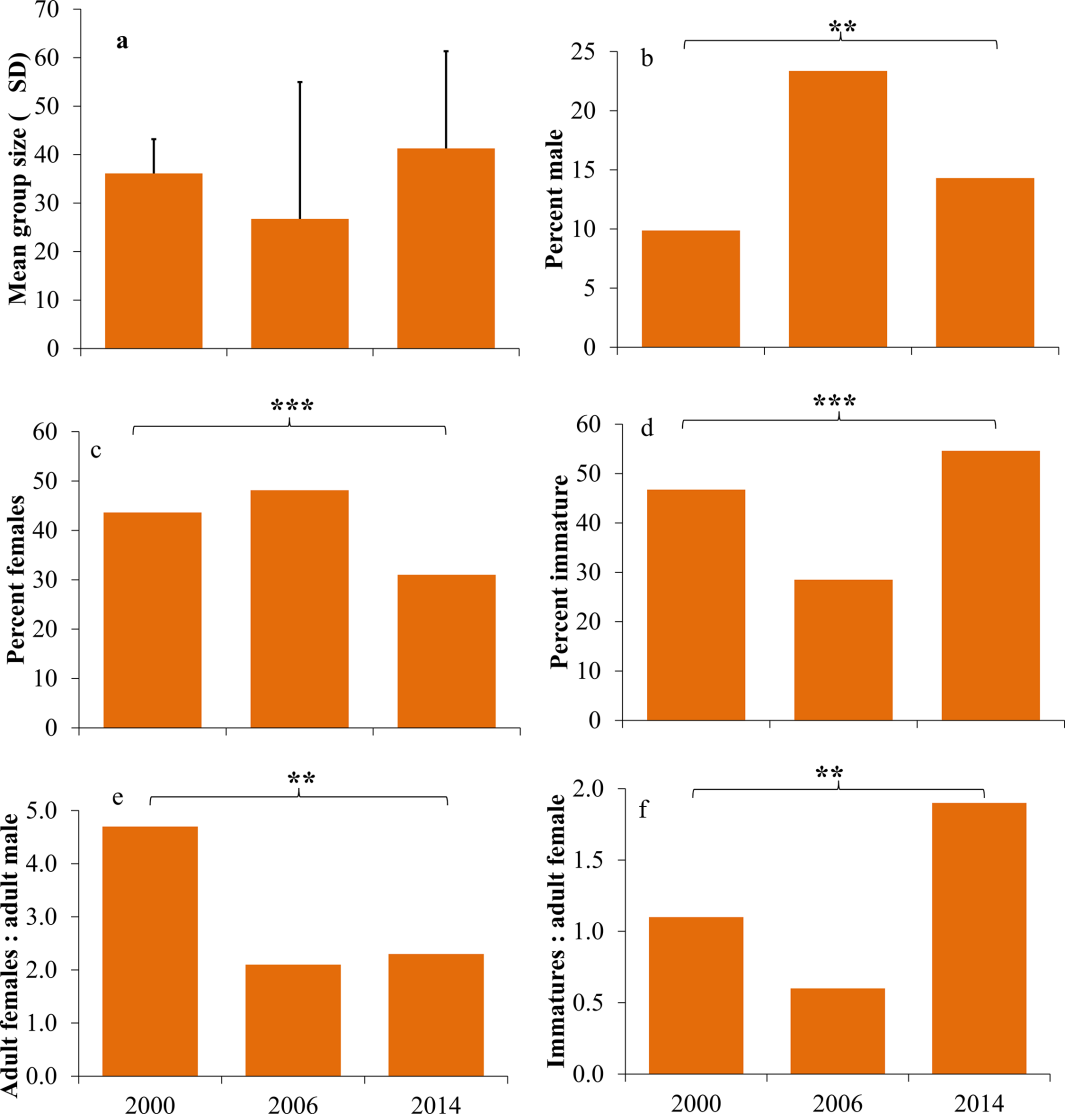
Comparison of group structure and age- sex ratios of *M*. *f*. *Umbrosus* between three study periods. a) mean group size, b) percent males, c) percent females, d) percent immature, e) females per adult male, f) immature per adult female. **p<0.01; ***p<0.001.

Although statistically not significant (F_2, 23_ = 1.057, p = 0.36), the mean group size declined from 36.12 ±7.07 in 2000 to 26.75 (±28.23) by 2006, and increased to 41.30 ± 20.02 by 2014 (Fig 3A).Thepercentof adult males, adult females and immatures per group differed significantly across thethree surveys (Adult males F_2, 23_ = 9.45, p = 0.01; adult females F_2, 23_ = 10.41, p = 0.01; and immatures F_2, 23_ = 17.20, p = 0.01) (Fig 3B–3D). Percent adult males and females per group increased from 9.87 and 43.63 in 2000 to 23.36 and 48.13 by 2006, and decreased to 14.30 and 31.00 by 2014 respectively. Conversely, percent immature per group decreased from 46.75 in 2000 to 28.50 by 2006, and increased to 54.60 by 2014. The number of adult females to adult males has significantly decreased from 4.74 in 2000 to 2.06 by 2006 and 2.32 by 2014 (F_2, 23_ = 7.43, p = 0.01) (Fig 3E). The immature to adult female ratio significantly decreased from 1.09 in 2000 to 0.59 by 2006 and again increased to 1.89 by 2014 (F_2,23_ = 12.59, p = 0.01) (Fig 3F).

## Discussion

We walked 212.14 km and sighted 79 groups of *M*. *f*. *umbrosus*. The encounter rate of groups per kilometre was higher in Katchal than in other islands, in plantations and mixed forests than in other forest types andin coastal areas than in inland habitat. Group structure of monkeys did not vary among islands except for Katchal having significantly more immature*s* than Great Nicobar. The Tsunami affected the western coast more than the eastern coast. Population*s* of *M*. *f*. *umbrosus*, which had declined immediately after the tsunami, had recovered and became more in 2014 than the pre-tsunami populations. Despite the current population sizebeing higher than pre-tsunami conditions, we found significant changes in group structure and composition.

ATsunami is a category ‘I’ disaster, which occurs for a short duration but has asevere impact. Apart from direct mortality of individuals, it caused heavy damage to coastal habitats which in turn affected the population. Our study reveals that Katchal (13.52%) had lost proportionally *larger* area than Little Nicobar (6.77%) and Great Nicobar (5.48%). Porwal et.al.[33]and Ramachandran et.al. [32]reported similar findings of severe damage to littoral forests, mangroves and low land swamps in all these three islands, and further high intensity of damage in Katchal. This has lead the people to leave Katchal or shift their residence by abandoning many coconut and palm plantations to other areas.

Although *M*. *fascicularis* is known to refuge in riverine and coastal regions[27,28,39], they are highly adaptable and adjusttheir activity pattern to changing conditions even by exploiting novel food sources[12,24]. Due to their phenotypic plasticity, speedy recovery of the population was expected. Umapathy et.al.[29]reported thepresence of population*s* in the entire island including inland evergreen forests. Populations in unaffected inland forests could have facilitated re-colonization of *M*. *f*. *umbrosus* in all the three islands of Nicobar. Despite thepopulation of *M*. *f*. *umbrosus* suffering adrastic decline followingthe tsunami[15], they have shown an increase in population size, especially in Katchal Island which had the lowest abundance in previous studies. Umapathy et.al.[30]reported minimal level of crop raiding in certain areas.However, during our interaction with the local people in Great Nicobar and Katchalthey reported increased crop raiding (coconut and banana) by macaques in villages after thetsunami. This indicates that they have adapted to new food sources. Indeed, *M*. *f*. *umbrosus* has shown a high degree of recovery of population size. This is probably due to increased resource availability from abandoned coconut, oil palm plantations, and adapting to feed on different agriculture crops. This may be the reason why Katchal Island shows increased abundance despite the fact that it has lost proportionally larger area.

Different species might adopt different strategies to overcome populationdecrease. One of the strategies may be increasing their population size by increasing the reproductive rate. Although the mean group size did not differ between the three surveys, it showed considerable increase by 2014 and altered the demographic structure. Disasters affected different age-sex classes differently by increasing mortality of weaker individuals in ottarids and ring tailed lemurs [3,6]. In *M*. *f*. *umbrosus*,thenumber of females per adult males decreased from 5 to 2(ratio 4.74 to 2.32), while the number of immatures per adult females increased from 1 to 2 (ratio 1.09 to 1.89). Increased adult males per group and decreased ratios of adult female*s* to adult males may be due to decreased habitat and total space availability for thedispersal of males. Dittus [40] reported that after a bad storm, reduced the food supply for toque macaques in Sri Lanka, adult males out competed adult females for what food remained and that both outcompeted immature, ultimately decreasing the number of immature individuals. In our study, increased numbers of immatures indicates increased reproductive rate, which may be the strategic response to asudden decline in the population size which is another indicator of population recovery.

## Conclusion

This is a case study on a terrestrial species, which documented the recovery of the population following asharp decline due to catastrophic event;a tsunami in an island system. This also reveals that some speciescan adapt by changing their ecology and behaviour or rates of infant survivorship.Although this study indicates populationrecovery based on the available information, stability of the population cannot be ascertained. Various sophisticated statistical techniques available today can make use of partially observed data and incorporating stochasticity, and can predict the extinction risk for the population. But for species like *M*. *f*. *umbrosus* which inhabit remote islands, it becomes logistically difficult to obtain the data required for stochastic modelling. A regular monitoring of species with restricted ranges such as island dwelling species is required, since such species are more prone to extinctions due to tsunamis and other natural disasters, due to their isolation and restricted geography.

## Supporting Information

S1 Table Details of the trail survey conducted. (XLSX)
